# Age-Related Onset of Obesity Corresponds with Metabolic Dysregulation and Altered Microglia Morphology in Mice Deficient for Ifitm Proteins

**DOI:** 10.1371/journal.pone.0123218

**Published:** 2015-04-09

**Authors:** Yin Shen Wee, Janis J. Weis, Lorise C. Gahring, Scott W. Rogers, John H. Weis

**Affiliations:** 1 Department of Pathology, Division of Microbiology and Immunology, University of Utah School of Medicine, Salt Lake City, Utah, United States of America; 2 Geriatric Research, Education and Clinical Center (GRECC), Veterans Affairs Medical Center, Salt Lake City, Utah, United States of America; 3 Department of Internal Medicine, Division of Geriatrics, University of Utah School of Medicine, Salt Lake City, Utah, United States of America; 4 Department of Neurobiology and Anatomy, Salt Lake City, Utah, United States of America; University of Cordoba, SPAIN

## Abstract

The *IfitmDel* mouse lacks all five of the *Ifitm* genes via LoxP deletion. This animal breeds normally with no obvious defect in development. The *IfitmDel* animals exhibit a steady and significantly enhanced weight gain relative to wild-type controls beginning about three months of age and under normal feeding conditions. The increased weight corresponds with elevated fat mass, and in tolerance tests they are hyporesponsive to insulin but respond normally to glucose. Both young (4 mo) and older (12 mo) *IfitmDel* mice have enhanced levels of serum leptin suggesting a defect in leptin/leptin receptor signaling. Analysis of the gene expression profiles in the hypothalamus of *IfitmDel* animals, compared to WT, demonstrated an altered ratio of *Pomc* and *Npy* neuropeptide expression, which likely impairs the satiation response of the *IfitmDel* animal leading to an increased eating behavior. Also elevated in hypothalamus of IfitmDel mice were pro-inflammatory cytokine expression and reduced IL-10. Anatomical analysis of the hypothalamus using immunohistochemistry revealed that microglia exhibit an abnormal morphology in *IfitmDel* animals and respond abnormally to Poly:IC challenge. These abnormalities extend the phenotype of the IfitmDel mouse beyond abnormal responses to viral challenge to include a metabolic phenotype and weight gain. Further, this novel phenotype for the *IfitmDel* mouse could be related to abnormal neuropeptide production, inflammatory status and microglia status in the hypothalamus.

## Introduction

The interferon-induced transmembrane gene family (Ifitm) consists of four genes in humans and five in the mouse that encode very similar proteins of 40–60 residues. Each Ifitm protein consists of a unique extracellular N-terminus, a highly conserved transmembrane domain, and equally well-conserved hydrophilic (cytoplasmic) domain followed by a much more diverse transmembrane-like domain [1]. Although the Ifitm proteins can be detected with sequences on the outside of the plasma membrane, an alternative prediction is that they are embedded on the cytoplasmic side of the membrane [2]. The proteins possess a number of functional sites including cysteine residues that are palmitoylated, lysine residues that are ubiquitinylated and serine/threonine residues that are phosphorylated following cellular activation ([1,2],unpublished data). Ifitm cell location includes on the surface as well as inside the cell where they are associated with endosomal and golgi membranes [3–5]. As a class, the Ifitm proteins have a proclivity to bind to multi-spanning/tetraspanin proteins such as CD81 and CD9 [6,7].

Of the Ifitm gene family members, Ifitm3 is the best characterized and it shows the greatest transcriptional response to type I and type II interferon induction[8]. Ifitm3 was shown in a broad siRNA screen to be essential for the interferon-induced cellular resistance to viruses that infect from the endosomal compartment to the cytoplasm such as influenza and dengue [4,5,9,10]. A defective human IFITM3 allele has been linked to increased severity of human infections to influenza virus [11] and we have recently shown this same allele is linked to coronary heart damage associated with Kawasaki Disease, an immune inflammation of unknown initiation [12]. A number of models have been proposed to describe the function of Ifitm3 in providing resistance to cellular infections including building a protein lattice in the membrane to block endosomal exit, blocking fusion pores during virus-endosome hemifusion, enhancing the deposition of cholesterol to also block virus exit or by blocking virus entry by enhancing the stability of the clathrin/vATPase complexes on the endosomal membrane [13–15].

The mouse Ifitm gene family encompasses about 65,000bp on mouse chromosome 7. This section of the chromosome has been removed by LoxP mediated deletion to create the *IfitmDel* animal which lacks all five of the Ifitm genes [16]. No other coding sequences or functional non-coding RNA’s are included within this section of the genome. The *IfitmDel* animal was originally created to test the necessity of the Ifitm proteins for germinal cell speciation [17–19] and embryo generation [20]. *IfitmDel* animals are generated in normal Mendelian numbers and have few if any obvious defects in development and survival [16]. We have made extensive use of these animals to study the roles that the Ifitm proteins have in immune signaling pathways. As we maintain these animals as homozygous deletion lines, over time we have observed a pronounced enhanced weight gain and an obesity phenotype (e.g., [21–23]) in older *IfitmDel* mice compared to C57BL/6 controls. In this report we quantify the obesity phenotype and link this to altered leptin/neuropeptide signaling, and demonstrate abnormal microglia morphology in the *IfitmDel* animal.

## Materials and Methods

### Animals

The mice were housed and used for this study in accordance with protocols approved in advance by the Institutional Animal Care and Use Committee at the University of Utah (Protocol Number (09–07003). In all cases animals were maintained in according to the Guide for the Care and use of Laboratory Animals of the National Institutes of Health. *IfitmDel* mice were backcrossed for greater than 10 generations to the C57BL/6 strain. Background- and age-matched littermates were used as WT controls. These mice were fed ad libitum with normal chow. For food intake studies, mice were kept individually and a similar amount of normal chow was given to each mouse. Average three-day consumption of food was measured for 21 days.

### Metabolic studies

For insulin and glucose tolerance tests, mice fasted 5 hours followed by *intraperitoneal injection* of human recombinant insulin (1U/kg, Novalin R) or glucose (1.5g/kg, Sigma) respectively. Blood levels were measured at indicated time points by *FreeStyle Lite* blood glucose monitoring system. Blood samples were obtained from tail bleeds.

For blood tests of fasting and fed animals, including blood glucose and leptin, blood was obtained at 10 am and 10 pm (12h) for analysis.

Energy expenditure and locomotor activity were analyzed by indirect colorimetry using CLAMS metabolic cages, HSC Cores Research Facility at University of Utah. Body composition was determined by nuclear magnetic resonance (NMR Bruker Minispec).

### RNA isolation and gene expression analysis

For RNA isolation, the hypothalamus was dissected, total RNA of the hypothalamus extracted by using QAIzol reagent kit (Qaigen), and reverse transcribed with Maloney Murine Leukemia Virus Reverse Transcriptase (M-MLV RT, Invitrogen). For qRT-PCR, 1x FastStart Universal SYBR Green Master (Roche) was used to analyze gene expression on LightCycler 480 System (Roche). The accession numbers of genes *Ifitm1* (NM_026820.3), *Ifitm2* (NM_030694), *Ifitm3* (NM_025378), *Ifitm6* (NM_001033632.1), *NPY* (NM_023456.2), *POMC* (NM_001278581), *TNFα* (NM-013693), *IFNβ* (NM_010510), *Ifit1* (NM_008331), *iNOS* (NM_010927) *IL-10* (NM_010548) *IL-1β* (NM_008361), *F4/80* (NM_010130) and *actin* (M12866.1). Primer sequences were as follows: *Ifitm1*, forward: 5’-CTTCAAAAGCCGAGAGATG-3’, reverse: 5’-CCACCATCTTCCTGTCCCTA-3’; *Ifitm2*, forward: 5’-CCATCCTCCAGACGGGGCGATTG-3’, reverse: 5’-TATTCAGGCACTTGGCAGTG-3’; *Ifitm3*, forward: 5’-CTTTGCTCCGCACCATGAACCA-3’, reverse: 5’-AGGCACTTAGCAGTGGAGGCGT-3’; *Ifitm6*, forward: GAGGGATCCTGACTCAGC-3’, reverse: 5’-AGCATGGGATTGGGCCCCAGTC-3’; *POMC*, forward: CTGCTTCAGACCTCCATAGATGTG-3’, reverse: 5’-CAGCGAGAGGTCGAGTTTGC-3’; *NPY*, forward: 5’-TACTCCGCTCTGCGACACTA-3’, reverse: 5’-GATGAGGGTGGAAACTTGGA-3’; *actin*, forward: 5’-GTAACAATGCCATGTTCAAT-3’, reverse: 5’-CTCCATCGTGGGCCGCTCTAG-3’; *IFNβ*, forward: 5’-CAAGAAAGGACGAACATTCG-3’, reverse: 5’-AGACATTCTGGAGCATCTCT-3’; *Ifit1*, forward: 5’-ATGGGAGAGAATGCTGATGGTG, reverse: 5’-TGTCAAGGAACTGGACCTGCTC-3’; TNFα and IL-1β [24], IL-10, [25] F4/80 [26] and iNOS [27] can be found in indicated citations.

### Histology, immunohistochemistry and microscopy

The epididymal fat pads were dissected from animal and fixed immediately in 4% paraformaldeyde in PBS overnight at 4°C. Next day, tissues were dehydrated with serial alcohol solutions (50%, 70% and 100%) at room temperature for 2 hours each. Tissues were then infiltrated in Immuno-bed resin (Polyscience, Inc.) at room temperature overnight. Tissues were moved to fresh Immuno-bed resin with catalyst (Polyscience, Inc.) at 1:25 ratio and polymerized in the mold at room temperature overnight. Tissues were carefully removed from the mold and sectioned to 3 microns with a rotary microtome (Thermo Scientific, Microm HM310). Tissue sections were adhered to microscope slides and proceed to H&E staining according to manufacturer's instructions (VWR). Stained slides were mounted with cytoseal mounting medium (Fisher Scientific) before imaging. Pictures were acquired with Zeiss Axiovert 100 microscope equipped with a Microfire CCD camera (Optimetrix). Brain sections were prepared as before [28–30]. Briefly, mice were lethally anesthetized with tribromoethanol (TBE) by intraperitoneal injection. For perfusion, the right ventricle of the heart was punctured and 10mL of saline was perfused into the left ventricle, followed by 20mL of 3% paraformaldehyde (PFA, EMS) plus 5% sucrose in PBS. The brains were post-fixed with 3% paraformaldehyde plus 5% sucrose solution and subsequently infiltrated with 15% and then 30% sucrose. Brain tissues were embedded in 2% gelatin (Sigma–Aldrich) and sectioned at 10μm with the Thermo Scientific HM 550 Cryostat (ThermoFisher Scientific). Sections were permeabilized with 0.02% Triton (Sigma) and stained with Iba1 (Abcam), F4/80 (eBioscience), CD11b (eBioscience), GFAP (Abcam), O2A (Abcam) and MAP-2(Abcam) followed by secondary antibody FITC- or PE-conjugated anti-rabbit antibodies or Alexa Fluor 546 Goat Anti-Rat IgG (invitrogen) as appropriate.

BMDM were isolated and cultured using standard protocols (14). Cells were fixed with 4% paraformaldehyde and permeabilized with 0.02% Triton (Sigma) and stained with Iba1.

## Results

### The *IfitmDel* animals demonstrate enhanced adiposity

**Fig 1:**
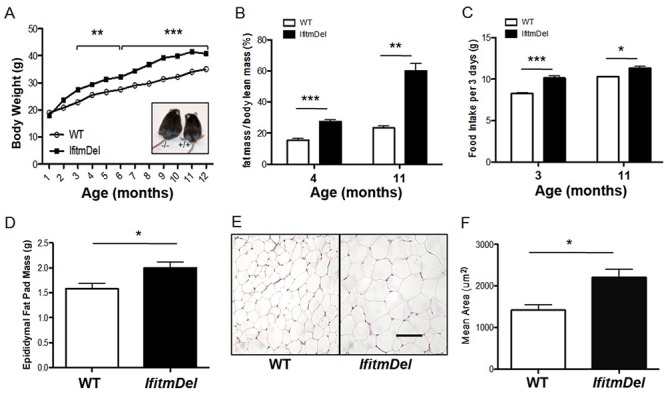
Deletion of *Ifitm* gene family leads to obesity. (A) Weight of male mice on normal chow, *IfitmDel*: Shown is the representative weight of wild-type (n = 10–22 per point) and *IfitmDel* (n = 4–16 per point). Both are in the C57BL/6 genetic background. Error bars were not included for clarity. (B) Fat mass was analyzed by nuclear magnetic resonance (4-month-old, n = 4 per group; 11-month-old is: wt: n = 3, *IfitmDel*: n = 4). (C) Food intake was measured every three days and over a period of three weeks for each group (4-month-old n = 4 for each group; 11-month-old wt: n = 3, *IfitmDel*: n = 4). (D) The mass of epididymal fat depots of male mice (7–10 weeks; wt: n = 5, *IfitmDel*: n = 4). (E) Histological appearance of epididymal fat depots from 8-week-old mice taken from hematoxylin and eosin stained sections. Scale bar: 100 μm. (F) The mean area of individual adipocytes was determined from multiple sections of H&E stained sections as shown in Panel E. For all statistical comparisons the Student’s t-test was used to evaluate significance between like groups where * = p <0.05; ** = p <0.01; and *** = p<0.0001.

The *IfitmDel* strain possesses a defined and engineered (via Cre-Lox) chromosomal deletion of all five of the *Ifitm* genes on a mouse genetic background of C57BL/6[16]. No coding sequences or regulatory sequences other than those associated with the *Ifitm* genes are known to be lost in this deletion. Mice lacking these *Ifitm* genes are fertile and thrive in colonies except for the well-described sensitivity to viral infections. In maintaining such mice in our colony we noted that the older *IfitmDel* animals were generally larger than their WT (C57BL/6) age and sex matched counterparts. To quantify these differences, body weights were taken from male mice maintained in the colony on regular mouse chow. Fig 1A, shows the slow but significant enhanced weight gain associated with the *IfitmDel* animals compared to WT. This body weight increase corresponded with an increased total fat mass as the animals aged (Fig 1B). Measurement of 3 day food intake averages for a period of 21 days shows that the *IfitmDel* mice consume more chow than the WT age-matched controls (Fig 1C). Also increased levels of epididymal fat deposits were present in these (Fig 1D). Histological examination suggested the adipocyte size was increased compared to WT adipose tissue (Fig 1E) and this was confirmed by quantitation as shown in Fig 1F. The same trends were observed for female mice (not shown).

### The *IfitmDel* animals demonstrate metabolic alterations

**Fig 2:**
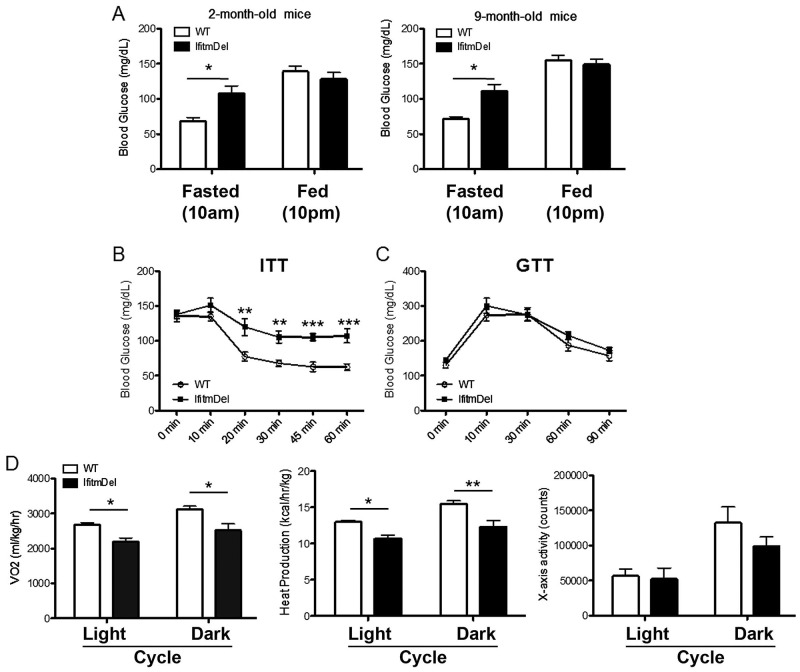
*IfitmDel* mice develop metabolic dysfunction. (A) Blood glucose of 2- and 9-month old WT and *IfitmDel* mice were measured at 10 AM (fasted) or 10 PM (fed). (B) & (C) Insulin tolerance test and glucose tolerance test of male mice (20–24 weeks; n = 7 for each group). (D) Leptin levels in the blood collected from tail veins were determined by ELISA. (E) Oxygen consumption, heat production and activity measured using metabolic cages (11-month-old male mice; n = 3 for each group). *p <0.05; **p <0.01; ***p<0.0001.

We next determined if the enhanced body weight of the *IfitmDel* animals was associated with altered physiological stasis. Blood glucose levels of fasting 2 month and 9-month-old male *IfitmDel* animals were measured and observed to be consistently higher than WT (Fig 2A). However, this difference was not observed under normal feeding (fed) schedules. This difference in fasting blood glucose was clear in the 2 month old animals.

Two standard tests to measure blood glucose levels are the insulin tolerance test (ITT) (Fig 2B) where blood glucose levels are quantified in fasted mice following a single injection of insulin and the glucose tolerance test (GTT; Fig 2C) in which blood glucose levels are quantified following a single injection glucose[31]. As shown, the *IfitmDel* animals responded poorly in the ITT, maintaining higher levels of blood glucose than the WT animals. The *IfitmDel* and WT animals were indistinguishable in their response in the GTT. The same trends were observed with female *IfitmDel* animals (not shown). These results suggest that as the *IfitmDel* animals age, they enter into a metabolic syndrome with a more moderate phenotype than animals displaying morbid obesity [23].

The *IfitmDel* animals were also analyzed using metabolic chambers that quantify oxygen consumption (V02), heat production, and activity (movement). As shown in Fig 2D the *IfitmDel* animals have reduced metabolism compared to the WT animals as reflected by lower oxygen consumption and decreased heat production in both the light and dark cycles. The activity of the *IfitmDel* animal in the cage trended towards less than the WT in the 12 month age group, but these differences were not statistically significant.

### The *IfitmDel* animals possess elevated levels of serum leptin

**Fig 3:**
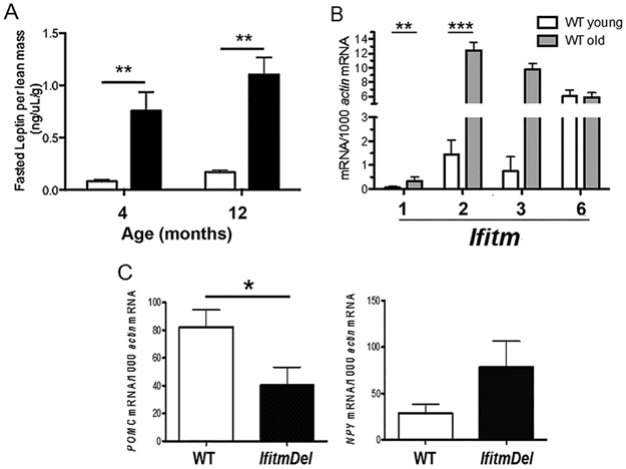
*IfitmDel* mice both show hyperleptinemia and alter levels of POMC and NPY transcripts. (A) Fasted leptin levels of 4-month-old or 12-month-old mice age matched WT (n = 3 per group) or *IfitmDel* mice (n = 4 per group) were compared. (B) RNA was isolated from the hypothalamus of WT young (2 months) or older (12 month) mice. The transcript levels of Ifitm1, Ifitm2, Ifitm3 and Ifitm6 were then determined using quantitative PCR as described in methods and the results normalized for transcript levels to copies expressed per 1000 actin mRNA. (C) Using the same RNA samples, transcript levels of POMC or NPY were analyzed in a similar manner. *p <0.05; **p <0.01; ***p<0.0001.

Mammals with a metabolic syndrome phenotype often have altered levels of leptin in the blood stream [32]. The leptin cytokine is critical in regulating appropriate food uptake [22,33,34] and we have shown (Fig 1C) that the *IfitmDel* mice have increased food intake. This level of food intake, however, is significantly less than observed with the *ob/ob* or *db/db* mice. To assess leptin levels in the *IfitmDel* and control mice, two sets of male animals (4 and 12 months) were analyzed (Fig 3A). In order to correct for the differences in body weight, serum leptin levels are shown as ng per μl per gram of lean body mass. As shown in Fig 3A, the blood leptin levels were dramatically elevated in the *IfitmDel* animal compared to WT. When blood leptin levels in mice of the same weight (e.g., *IfitmDel* vs WT weighing 35 g), but differing age, were compared the *IfitmDel* animals still consistently have higher levels of blood leptin compared to WT controls (not shown). Leptin is primarily produced by fat cells and serves, via the leptin receptor expressed in the hypothalamus, to regulate food intake [23,34,35]. Animals lacking leptin (*ob/ob*) or a functional leptin receptor (*db/db*) become morbidly obese due to their lack of control over food intake [34].

Leptin’s actions are largely through interaction with leptin receptors expressed in the hypothalamus, which makes this brain region a target for the interaction between leptin and Ifitm. To confirm that *Ifitm* genes are expressed by cells of the hypothalamus, the expression of *Ifitm1*, *Ifitm2*, *Ifitm3* and *Ifitm6* was quantified in the WT hypothalamus samples (*Ifitm5* is only expressed by osteoblasts). As shown in Fig 3B, *Ifitm2*, *Ifitm3* and *Ifitm6* are all expressed in WT tissue, with *Ifitm2* and *Ifitm3* showing dramatically elevated expression in older animals. Therefore, the correlation between the deletion of *Ifitm* genes and leptin levels occurs in both young and older mice, the older *IfitmDel* mice have the added impact of not having the normal increase in these proteins seen during the aging process.

The leptin receptor, expressed by neurons in the hypothalamus, signals through a Stat3 dependent pathway, controlling the expression of two contrasting neuropeptides[34]. These are the proopiomelanocortin (*Pomc*) gene whose expression is increased upon leptin signaling while the transcription of the neuropeptide y (*Npy*) gene is depressed [36–39]. Bio-active peptides from the Pomc protein serve to dampen eating while those of Npy have the opposite effect and promote food intake. The expression of *Pomc* and *Npy* gene transcripts were analyzed from hypothalami obtained from WT and *IfitmDel* animals of various ages from 1 to 12 months of age. As shown in Fig 3C, *IfitmDel* animals have significantly reduced *Pomc* transcripts than WT suggesting that the *IfitmDel* animals lack appropriate signals to depress eating. Further, the *IfitmDel* samples have trending higher levels of *Npy* transcripts compared to WT samples suggesting elevated Npy peptides may contribute to the positive signal to maintain feeding.

### *IfitmDel* mice demonstrated altered responses to chronic Type I interferon induction

**Fig 4:**
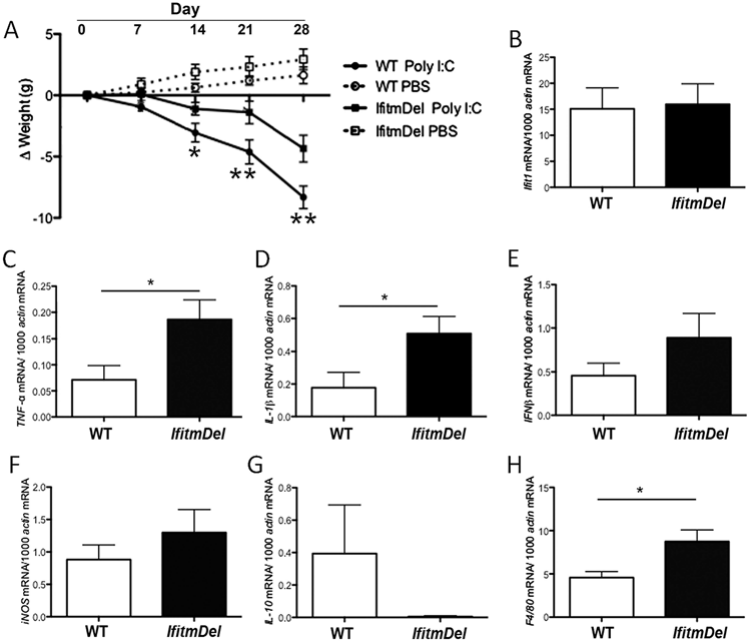
The *IfitmDel* mice exhibit an altered response to Poly I:C activation. Mice were injected with PBS or Poly I:C every two days for the 28 day course. (A) Changes in body weight after mice treatment with PBS or Poly I:C (12.5mg/kg) injection. (B-I) Comparison of hypothalamic gene expression of selected pro-inflammatory cytokines in WT versus *IfitmDel* mice after Poly I:C injection. Hypothalamus tissue was isolated after injection with PBS or Poly I:C every two days for 8 days of 6- to 8-week-old mice (n = 6 for each group). (B) Relative transcript levels of *Ifit1* (an interferon responsive gene unrelated to *Ifitm* genes) confirm the *IfitmDel* mice respond to this treatment regime similar to WT mice. Additional measurement of the expression of selected transcripts from the same sample as in panel B show varied responses of: (C) *TNFα*; (D) *IL-1β*; (E) *IFNβ*; (F) *iNOS*; (G) *IL-10*; and (H) *F4/80*. *p <0.05 and **p <0.01.

Chronic Poly I:C treatment, via activation of Tlr3 and the RIG-I-like receptors, results in the production of IL-6 and IFNγ that can, when provided chronically as a model for cachexia, lead to a progressive weight loss [40]. When administered *in vivo*, the major cell types responding to Poly I:C include macrophages, dendritic cells (DC) and microglia. Additionally responding DC’s can undergo necroptosis that can exacerbate the inflammatory response. Metabolic dysfunction and neurological disorders have also been linked to cachexia, especially as the outcome of chronic infections and cancer metastasis that can lead to the chronic release of inflammatory cytokines. Based upon the previously described findings that the absence of the Ifitm proteins can alter cellular induction pathways following type I interferon treatment, we tested whether or not the *IfitmDel* animals would have an altered response, compared to WT, to chronic Poly I:C treatment. As shown in Fig 4A, 8 week old WT animals treated with Poly I:C over a time course of 28 days demonstrated the expected progressive weight loss, but age and sex matched *IfitmDel* animals were much more resistant to the cachexic effects of the Poly I:C treatment. While the *IfitmDel* animals treated with Poly I:C did lose weight, especially compared to their PBS-treated counterparts, the degree of weight loss was much less dramatic. To determine if the altered response to Poly I:C in the *IfitmDel* animals was also mirrored in altered cytokine responses, animals were treated with Poly I:C for 10 days (treatment every two days) weighed and analyzed. The hypothalamus was removed by dissection, total RNA isolated and cytokine expression by quantitative RT-PCR was measured. As a positive control, the IFN-induced protein with tetratricopeptide repeats (Ifit1), which is genetically and functionally distinct from the Ifitm proteins, was measured in response to type I interferon stimulated by Poly I:C injection. As shown in Fig 4B, WT and *IfitmDel* mice have equivalent levels of Ifit1 RNA following Poly I:C treatment indicating these two strains were equally responsive. However, the *IfitmDel* and WT animals displayed significant differences in *TNFα* and *IL-1β* mRNA levels with the *IfitmDel* animal showing significantly elevated levels of these inflammatory cytokines (Fig 4C and 4D). PBS mock activated mice of the WT and *IfitmDel* did not induce elevated cytokine levels in the hypothalamus (not shown). The expression of the *IFNγ* and *iNOS* genes was also elevated in the *IfitmDel* animals (Fig 4E and 4F) with differences trending toward significance. Interestingly the expression of the anti-inflammatory cytokine IL-10 showed lower levels of expression in the *IfitmDel* animal samples (Fig 4G). Finally the expression of F4/80 (*Emr1*), a macrophage/microglial marker, was compared between the WT and *IfitmDel* Poly I:C treated animals (Fig 4H). The increased level of F4/80 RNA in the hypothalamus of the Poly I:C treated *IfitmDel* animals could be for a number of reasons including enhanced recruitment of blood monocytes/macrophages to that anatomic site compared to similarly activated WT animals. Similar analyses were performed with RNA obtained from abdominal fat pads (not shown) which showed the same trend of inflammatory cytokine signature as that obtained from the hypothalamus samples. In total these cytokine profiles suggest the response to interferon induced by the Poly I:C treatment is intact but much more polarized towards a pro-inflammatory signature in the *IfitmDel* animal than WT. This is the case even though the weight loss in these animals upon Poly I:C treatment is less severe.

### Immunohistochemistry of the WT and *IfitmDel* mice hypothalamus

**Fig 5:**
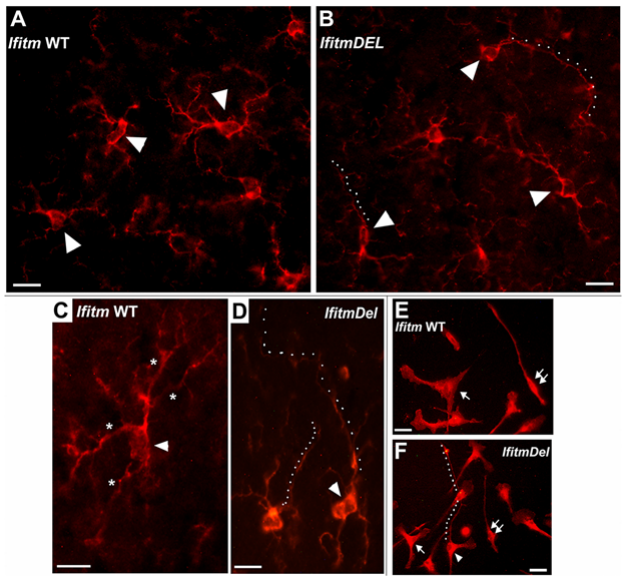
Microglia from *IfitmDel* mice exhibit morphologic abnormalities. (A) Immunohistochemical examination of sections prepared from saline perfused and paraformaldehyde fixed brains stained for the expression of the microglial marker, Iba1 (red), are shown. (A,B) Comparison of microglia from photographs taken of the lateral hypothalamic nucleus (region of the ventromedial nucleus shown). Arrow heads point to typical appearance of (A) microglia in the *Ifitm* wild-type (WT) mice or (B) *IfitmDel* mouse. Dotted lines indicate extending processes of microglia seen in IfitmDel mouse sections. Bar = 30 microns. (C,D) increased magnification of individual microglia as marked by an arrow head. In (C) *Ifitm* WT microglia exhibit numerous bifurcations (some indicated by asterisks) whereas (D) *IfitmDel microglia* show few bifurcations and the long extended processes (dots) with few bifurcations. Bars are 20 microns. (E,F) Cultured bone marrow-derived macrophages from (E) *Ifitm* WT or (F) *IfitmDel* mouse (Methods and text). The arrow points to a common cell morphology where cells are flattened and have fan-shaped lamella. The double arrow identifies a cell with an elongated cell morphology that also common to cultures prepared from either genotype. Both are typical to both preparations. The arrow head identifies a cell with morphology unique to the *IfitmDel* culture that is characterized by exceptionally long thin processes (dots) that resemble the microglia morphology seen in the brain. Bar = 20 microns.

The RNA signaling results (Fig 4) led us to examine the morphology of the hypothalamus which to our knowledge has not been examined in depth in the *IfitmDel* mice. We first assessed the hypothalamus in unstimulated mice using markers for microglia/macrophages (Iba1), astrocytes (GFAP), oligodendrocytes (O2A) and neurons (MAP2). Coronal serial sections from the brains of saline perfused and paraformaldehyde-fixed tissue of *IfitmDel* and control animals (Methods) were prepared from equivalent anatomical locations, stained for cell specific markers and photographed for visual analysis. Overall no gross abnormalities in brain anatomy were detected (data not shown), which is consistent with earlier reports [16] that the *IfitmDel* animals exhibited no developmental defects (although CNS morphology was not specifically characterized). The regional distribution, appearance and staining of neurons, astrocytes or oligodendrocytes was also equivalent between animals of both genotypes (Methods). However, microglia revealed by immunostaining for the expression of the Iba1 antigen exhibited a markedly altered morphology between WT and *IfitmDel* mice (Fig 5). Among the most notable morphology differences was the occurrence of dramatically elongated processes that were often observed to extend well over 150 microns (Fig 5B, dotted line). These processes were almost exclusively unipolar, and they exhibited infrequent branching and few varicosities. The cell bodies of these microglia also tended to appear as smaller and more poorly defined relative to controls in the hypothalamus (Fig 5A–5D). The altered microglia morphology of the *IfitmDel* mice was evident in other brain regions including the cortex and hippocampus (data not shown). Microglia are mesoderm/mesenchymal derivatives that share a bone-marrow macrophage lineage [41]. To determine if the morphological differences observed in the *IfitmDel* microglia are intrinsic to this cell type, bone marrow macrophages (BMDM) from controls and *IfitmDel* mice were prepared from culture. After 10 days, cells were gently rinsed with PBS, cells fixed with paraformaldehyde and immunostained to reveal Iba1 expression. As shown in Fig 5E and 5F, the morphology of a subpopulation of *IfitmDel* BMDMs exhibited strikingly similar features to those seen in the microglia. In particular, these BMDMs from *IfitmDel* BM produced very thin extensions that extended for long distances in the cultures. These processes were rare or not detected with the control cells (Fig 5E). Also evident is the impression that *IfitmDel* BMDMs were more compact and in general looked smaller than cells of similar morphology in the WT preparation. This is also evident in cells producing fan-shaped lamella which are more compact in *IfitmDel* BMDMs. Thus the curious morphology of the *IfitmDel* microglia was recapitulated in cultured bone marrow derived macrophages possessing the same genetic defect suggesting that common cellular abnormalities may be associated with the *IfitmDel* in vulnerable cells.

**Fig 6:**
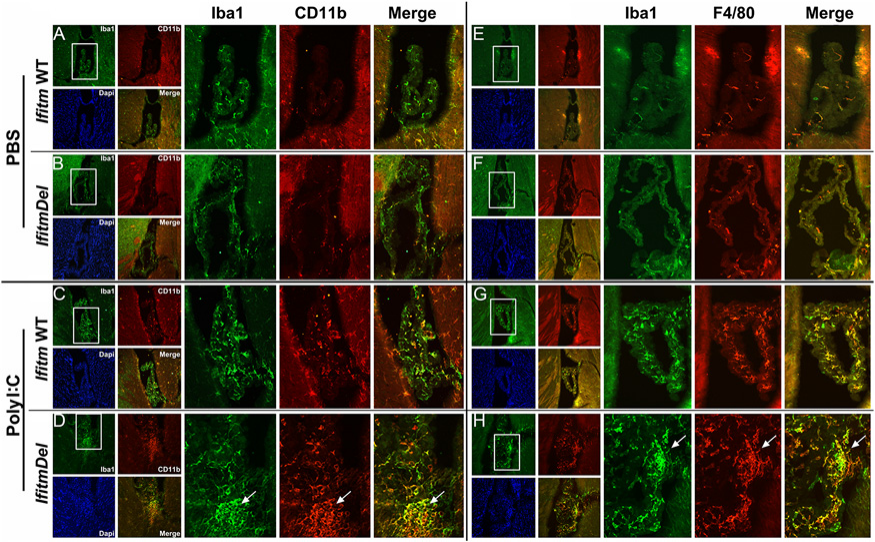
Microglia accumulation in the choroid plexus differs in *IfitmDel* mice treated with Poly I:C. Sections of the indicated genotype were prepared from the brains of mice treated with either PBS or Poly I:C (12.5mg/kg) injection as indicated. Shown are typical results of double labeling with either Iba1 (green), CD11b (red; A-D), or F4/80 (red; E- H), respectively. At low magnification (small panels; original magnification of 10x) and the DAPI image staining for cell nuclei of the same sections is included. Regions showing the choroid plexus (lateral ventricle; Bregma -0.1 to -0.22) are boxed and shown at greater magnification in the associated images. Images in (A and E) and (B and F) are staining of serial sections separated by approximately 50 microns. The arrows identify in the often large aggregates of stained cells unique to *IfitmDel* mice for either (D) Iba1/CD11b or from a different mouse (H) Iba1/F4/80. These aggregates are not present in similar sections of *Ifitm* WT mice (compare with (C) and (G), respectively). Similar results are common in choroid plexus of all ventricles (data not shown).

**Fig 7:**
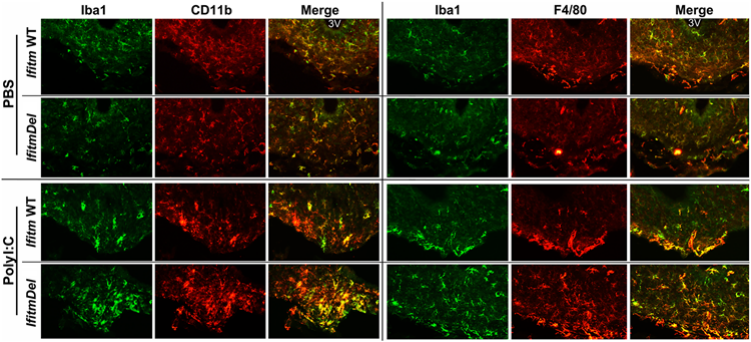
Microglia accumulation in the median eminence and ventral hypothalamus differs in *IfitmDel* mice treated with Poly I:C. Coronal sections prepared as described in the Fig 6 are shown for the region of the median eminence adjacent to the ventral hypothalamus (arcuate nucleus; approximate Bregma -1.58 to -1.7). Similar to the choroid plexus, increased accumulation of either Iba1/CD11b or Iba1/F4/80 cells are found in the PolyI:C treated *IfitmDel* mice. 3V = third ventricle.

Having found that microglia exhibit altered morphology in *IfitmDel* animal and the hypothalamic transcriptional response to Poly I:C is also altered in this mouse (Fig 4), we examined by immunohistochemistry changes in the CNS induced by Poly I:C. WT and *IfitmDel* animals were activated with Poly I:C as above, sacrificed and perfused. A notable difference in the response of the WT compared to the Poly I:C treated mice was evident in choroid plexus structures within ventricles. Macrophages mobilize to the CNS upon inflammation and this is often evident by increased cellularity of the choroid plexus. In Fig 6 we show that Poly I:C treatment of WT and *IfitmDel* animals stimulates an increase in the accumulation of Iba1/CD11b positive cells (Fig 6C and 6D; i.e., macrophages) in the choroid plexus. Further, these cells are also Iba1/F4-80 positive (Fig 6G and 6H), indicative of activation of macrophage lineage cells. A striking difference between WT and *IfitmDel* animals is the appearance of masses of these cells (Fig 6D and 6H) that are not evident in the PBS animals (Fig 6A and 6B), Poly I:C treated WT animals (Fig 6E and 6F) or the PBS-treated *IfitmDel* animal (Fig 6C and 6D). Similar data were also obtained from stained sections of the median eminence of the ventral hypothalamic region (Fig 7), again showing enhanced macrophage/microglia staining in the Poly I:C treated *IfitmDel* animal compared to controls or sham activated *IfitmDel* animals. Collectively these findings suggest that the enhanced numbers of transcripts encoding for F4/80 in the hypothalamus of Poly I:C treated *IfitmDel* animals (see Figs 6H and 7H) may be due to increased recruitment and/or accumulation of inflammatory macrophages to the brain. Further, the activated macrophages in these regions at the site of blood-brain interfaces suggests that perhaps the macrophages do not enter the brain but may become entangled in these regions during normal migration.

## Discussion

This report describes novel peripheral and central alterations associated with the lack of the Ifitm proteins. The *IfitmDel* animals lack all five of the *Ifitm* genes via an engineered genetic deletion without impact on other coding or control sequences that reside within the gene family locus. When bred as heterozygotes, the homozygous *IfitmDel* progeny are produced in the vivarium at normal Mendelian ratios and exhibit normal losses of the adult animals compared to WT C57BL/6 animals. What is clear, however, is that the longer the *IfitmDel* animals are maintained on normal chow diet, the more obese they become. This obesity is due to an enhanced accumulation of white adipose mass. We have not examined changes in brown adipose tissue in these animals.

While there are a variety of pathways that can lead to obesity, perhaps the best characterized are the aberrant feeding behaviors associated with alterations in the leptin/leptin receptor pathway [34]. Animals deficient in either the ligand or receptor become morbidly obese due to the uncontrolled feeding behavior of the animals. Leptin, produced by fat cells, binds to leptin receptor-bearing cells in the hypothalamus and engages the Stat3 signaling pathway to influence the expression of genes encoding neuropeptides [42]. These include genes that impact upon feeding behavior such as an increase in *Pomc* that suppresses feeding and *Npy* that promotes this activity. Hence, normally low leptin levels allow *Pomc* expression to drop and NPY to increase which leads to enhanced feeding. However, *IfitmDel* animals, even at an early age (1 month) with normal weight, despite increased leptin levels, exhibit depressed expression of *Pomc* that corresponds to the increased feeding behavior of these animals. This reveals a novel disparity in leptin signaling through its receptor in the hypothalamus. Our preliminary analyses of the Stat3 signaling pathways in the *IfitmDel* animals revealed no difference with WT animals (data not shown). Further, other signaling systems that require Stat3 activation such as the IL-6 receptor is similar in *IfitmDel* splenocytes to that of WT-responses (data not shown). However, the elevated expression of certain inflammatory cytokines such as TNFα, which is often associated with cachexia and weight loss, require further exploration in the IfitmDel mice. Thus, a future line investigation will be to detail the mechanism(s) through which the Ifitm proteins contribute to this unique metabolic phenotype.

Because we are unaware of any reports of brain structural anomalies for the *IfitmDel* animal, we performed a survey of brain using a histological approach to evaluate whether the altered *Pomc* expression regulation could be due to structural deficiencies in the hypothalamus. Overall, we observed no abnormal anatomical defects in any brain regions when compared to the C57BL/6 controls. Additional analyses of these sections stained with cell type specific immunofluorescent markers also failed to reveal any overall gross inconsistencies in the overall distribution or numbers of neurons, oligodendrocytes and astrocytes. Microglia, however, were different. The overall numbers appeared to be similar between the *IfitmDel* and control mice. It is worth noting that in occasional *IfitmDel* animals there appeared to be a substantial decrease in microglia in the cortex (not shown). What did consistently differ was a striking and common microglial cell morphology throughout the brain tissues of the *IfitmDel* animal. Most notable was the reduced elaboration of microglia morphology accompanied by extremely long and usually mono-polar processes that extended from the cell body with few bifurcations. Also, the cell bodies appeared smaller and less distinct than their control counterparts. How altered microglia cell morphology could specifically affect leptin signaling and the production of neuropeptides such as Pomc or Npy is not known. However, it is possible that IfitmDel microglia are unable to produce normal interactions with other cells types including neurons and astrocytes such as trophic interactions or clearance of debris thereby leading to altered and potentially toxic microenvironments.

The function(s) of the Ifitm proteins in development and maintenance of the central nervous system is a newly developing field. Elevated expression of *IFITM* family members has been noted in the brains of schizophrenic patients, patients with autism, bipolar disorders and Alzheimer’s disease [43–46]. Besides being reported in neurodegenerative diseases, *Ifitm3* also has been shown to respond to Poly I:C as an inducer of type I interferons, by increasing gene expression in astrocytes [47]. The Ifitm3 protein is found in the endosomes of astrocytes and knockdown of *Ifitm3* expression inhibits clathrin dependent uptake in such cells [47], similar to our description for cells obtained from the *IfitmDel* animal [14]. It appears that the expression of Ifitm proteins is crucial to function in astrocytes. However, our observations failed to reveal any gross morphological abnormalities in astrocytes of *IfitmDel* animals (stained by GFAP, data not shown). This will require further evaluation to assure astrocytes are not functionally compromised despite the overall appearance of normal morphology.

The dysregulation of the signaling pathways in regulating energy homeostasis in the hypothalamus leading to metabolic disorders are well-known. This includes neuronal dysfunctions or inflammation in certain brain regions may also be linked to metabolic disorders [48–50]. Activated microglia are capable of secreting pro-inflammatory cytokines that serve to recruit even more microglia to the site of inflammation that can further influence the metabolic response in the brain [51–54]. In neuronal dysfunctions such as schizophrenia, autism, bipolar disorders and Alzheimer’s disease [43–46], the activation of microglia in addition to astrocytes can worsen the disease progression by secreting excessive inflammatory cytokines. One by-product of these activation-dependent cytokines is the development of metabolic syndrome phenotypes characterized by weight gain and/or leptin resistance [52,55,56]. While there is a growing body of evidence suggesting the Ifitm proteins may influence neuronal function through poorly defined mechanism, our study suggests they actually play a role in normal brain cellular architecture and interaction.

A final point is that the *IfitmDel* animal is lacking all five of the *Ifitm* genes. As shown in Fig 4C, the *Ifitm1*, *2*, *3* and *6* genes are expressed in the hypothalamus and the expression of *Ifitm2* and *Ifitm3* normally increases with age. As the *IfitmDel* animal does not produce this age-related alteration in expression, there is the intriguing possibility that these increases produce normal compensatory functions towards control of the age-related increases in obesity and altered leptin modulation of metabolic homeostasis. Since an obesity phenotype has not been described for single *Ifitm* gene deletions (*Ifitm3* deficient or *Ifitm1* deficient)[16,53] nor have any brain anomalies such as the deficiency in microglia as shown in this report been described for any of these single *Ifitm* gene deletion strains, this will require further investigation. For example, *Ifitm6* is primarily expressed in osteoclasts and macrophage lineages (of which microglia are related [6,57]). But whether or not the microglia deficiency seen in the *IfitmDel* animal is due to the lack of *Ifitm6* during microglial cell development remains to be evaluated. Also other possibilities such as the indirect regulation of other modulators of adipose cell signaling by Ifitm (e.g., regulation of leptin signaling through carbonic anhydrase activation [58]) will need to be investigated. What does appear important is that *Ifitm* genes are implicated in modulating important endocrine functions. In the framework of the microglia and hypothalamic interactions, our data also suggest that the phenotype could vary depending upon exposure to *Ifitm*-specific pathogens.

## References

[1] Diamond MS, Farzan M. The broad-spectrum antiviral functions of IFIT and IFITM proteins. Nat Rev Immunol. 2013;3: 46–57. 10.1038/nri3344 PMC3773942 23237964

[2] Yount JS, Karssemeijer RA, Hang HC. S-palmitoylation and ubiquitination differentially regulate interferon-induced transmembrane protein 3 (IFITM3)-mediated resistance to influenza virus. J Biol Chem. 2012;287: 19631–19641. 10.1074/jbc.M112.362095 22511783 PMC3365998

[3] John SP, Chin CR, Perreira JM, Feeley EM, Aker AM, Savidis G, et al The CD225 domain of IFITM3 is required for both IFITM protein association and inhibition of influenza A virus and dengue virus replication. J Virol. 2013;87: 7837–7852. 10.1128/JVI.00481-13 23658454 PMC3700195

[4] Huang IC, Bailey CC, Weyer JL, Radoshitzky SR, Becker MM, Chiang JJ, et al Distinct patterns of IFITM-mediated restriction of filoviruses, SARS coronavirus, and influenza A virus. PLoS Pathog. 2011;7: e1001258 10.1371/journal.ppat.1001258 21253575 PMC3017121

[5] Feeley EM, Sims JS, John SP, Chin CR, Pertel T, Chen LM, et al IFITM3 inhibits influenza A virus infection by preventing cytosolic entry. PLoS Pathog. 2011;7: e1002337 10.1371/journal.ppat.1002337 22046135 PMC3203188

[6] Smith RA, Young J, Weis JJ, Weis JH. Expression of the mouse fragilis gene products in immune cells and association with receptor signaling complexes. Genes Immun. 2006;7: 113–121. 16395393 10.1038/sj.gene.6364278

[7] Matsumoto AK, Martin DR, Carter RH, Klickstein LB, Ahearn JM, Fearon DT. Functional dissection of the CD21/CD19/TAPA-1/Leu-13 complex of B lymphocytes. J Exp Med. 1993;178: 1407–1417. 7690834 10.1084/jem.178.4.1407 PMC2191213

[8] Jaffe EA, Armellino D, Lam G, Cordon-Cardo C, Murray HW, Evans RL. IFN-gamma and IFN-alpha induce the expression and synthesis of Leu 13 antigen by cultured human endothelial cells. J Immunol. 1989;143: 3961–3966. 2512344

[9] Chan YK, Huang IC, Farzan M. IFITM proteins restrict antibody-dependent enhancement of dengue virus infection. PLoS One. 2012;7: e34508 10.1371/journal.pone.0034508 22479637 PMC3316688

[10] Jiang D, Weidner JM, Qing M, Pan XB, Guo H, Xu C, et al Identification of five interferon-induced cellular proteins that inhibit west nile virus and dengue virus infections. J Virol. 2010;84: 8332–8341. 10.1128/JVI.02199-09 20534863 PMC2916517

[11] Everitt AR, Clare S, Pertel T, John SP, Wash RS, Smith SE, et al IFITM3 restricts the morbidity and mortality associated with influenza. Nature. 2012;484: 519–523. 10.1038/nature10921 22446628 PMC3648786

[12] Bowles NE, Arrington CB, Hirono K, Nakamura T, Ngo L, Wee YS, et al Kawasaki disease patients homozygous for the rs12252-C variant of interferon-induced transmembrane protein-3 are significantly more likely to develop coronary artery lesions. Mol Genet Genomic Med. 2014;2: 356–361. 10.1002/mgg3.79 25077179 PMC4113277

[13] Amini-Bavil-Olyaee S, Choi YJ, Lee JH, Shi M, Huang IC, Farzan M, et al The antiviral effector IFITM3 disrupts intracellular cholesterol homeostasis to block viral entry. Cell Host Microbe. 2013; 13: 452–464. 10.1016/j.chom.2013.03.006 23601107 PMC3646482

[14] Wee YS, Roundy KM, Weis JJ, Weis JH. Interferon-inducible transmembrane proteins of the innate immune response act as membrane organizers by influencing clathrin and v-ATPase localization and function. Innate Immun. 2012;18: 834–845. 10.1177/1753425912443392 22467717

[15] Desai TM, Marin M, Chin CR, Savidis G, Brass AL, Melikyan GB. IFITM3 restricts influenza A virus entry by blocking the formation of fusion pores following virus-endosome hemifusion. PLoS Pathog. 2014;10: e1004048 10.1371/journal.ppat.1004048 24699674 PMC3974867

[16] Lange UC, Adams DJ, Lee C, Barton S, Schneider R, Bradley A, et al Normal germ line establishment in mice carrying a deletion of the Ifitm/Fragilis gene family cluster. Mol Cell Biol. 2008;28: 4688–4696. 10.1128/MCB.00272-08 18505827 PMC2493357

[17] Tanaka SS, Matsui Y. Developmentally regulated expression of mil-1 and mil-2, mouse interferon-induced transmembrane protein like genes, during formation and differentiation of primordial germ cells. Gene Expr Patterns. 2002;2: 297–303. 12617817 10.1016/s0925-4773(02)00384-2

[18] Tanaka SS, Nagamatsu G, Tokitake Y, Kasa M, Tam PP, Matsui Y. Regulation of expression of mouse interferon-induced transmembrane protein like gene-3, Ifitm3 (mil-1, fragilis), in germ cells. Dev Dyn. 2004;230: 651–659. 15254899 10.1002/dvdy.20085

[19] Tanaka SS, Yamaguchi YL, Tsoi B, Lickert H, Tam PP. IFITM/Mil/fragilis family proteins IFITM1 and IFITM3 play distinct roles in mouse primordial germ cell homing and repulsion. Dev Cell. 2005;9: 745–756. 16326387 10.1016/j.devcel.2005.10.010

[20] Lange UC, Saitou M, Western PS, Barton SC, Surani MA. The fragilis interferon-inducible gene family of transmembrane proteins is associated with germ cell specification in mice. BMC Dev Biol. 2003;3: 1 12659663 10.1186/1471-213X-3-1 PMC153542

[21] Supuran CT, Di Fiore A, De Simone G. Carbonic anhydrase inhibitors as emerging drugs for the treatment of obesity. Expert Opin Emerg Drugs. 2008;13: 383–392. 10.1517/14728214.13.2.383 18537527

[22] Yang R, Barouch LA. Leptin signaling and obesity: cardiovascular consequences. Circ Res. 2007;101: 545–559. 17872473 10.1161/CIRCRESAHA.107.156596

[23] Zhang Y, Proenca R, Maffei M, Barone M, Leopold L, Friedman JM. Positional cloning of the mouse obese gene and its human homologue. Nature. 1994;372: 425–432. 7984236 10.1038/372425a0

[24] Crandall H, Dunn DM, Ma Y, Wooten RM, Zachary JF, Weis JH, et al Gene expression profiling reveals unique pathways associated with differential severity of lyme arthritis. J Immunol. 2006;177: 7930–7942. 17114465 10.4049/jimmunol.177.11.7930

[25] Miller JC, Ma Y, Bian J, Sheehan KC, Zachary JF, Weis JH, et al A critical role for type I IFN in arthritis development following Borrelia burgdorferi infection of mice. J Immunol. 2008;181: 8492–8503. 19050267 10.4049/jimmunol.181.12.8492 PMC3024833

[26] Brown CR, Lai AY, Callen ST, Blaho VA, Hughes JM, Mitchell WJ. Adenoviral delivery of interleukin-10 fails to attenuate experimental Lyme disease. Infect Immun. 2008;76: 5500–5507. 10.1128/IAI.00808-08 18824530 PMC2583579

[27] Ma Y, Seiler KP, Tai KF, Yang L, Woods M, Weis JJ. Outer surface lipoproteins of Borrelia burgdorferi stimulate nitric oxide production by the cytokine-inducible pathway. Infect Immun. 1994;62: 3663–3671. 7520417 10.1128/iai.62.9.3663-3671.1994 PMC303016

[28] Gahring LC, Persiyanov K, Dunn D, Weiss R, Meyer EL, Rogers SW. Mouse strain-specific nicotinic acetylcholine receptor expression by inhibitory interneurons and astrocytes in the dorsal hippocampus. J Comp Neurol. 2004;468: 334–346. 14681929 10.1002/cne.10943

[29] Gahring LC, Rogers SW. Nicotinic acetylcholine receptor expression in the hippocampus of 27 mouse strains reveals novel inhibitory circuitry. Hippocampus. 2008;18: 737–749. 10.1002/hipo.20430 18446824 PMC2792088

[30] Rogers SW, Weis JJ, Ma Y, Teuscher C, Gahring LC. Mouse chromosome 11 harbors genetic determinants of hippocampal strain-specific nicotinic receptor expression. Hippocampus. 2008;18: 750–757. 10.1002/hipo.20454 18528848 PMC2775497

[31] Zinker BA, Rondinone CM, Trevillyan JM, Gum RJ, Clampit JE, Waring JF, et al PTP1B antisense oligonucleotide lowers PTP1B protein, normalizes blood glucose, and improves insulin sensitivity in diabetic mice. Proc Natl Acad Sci USA. 2002;99: 11357–11362. 12169659 10.1073/pnas.142298199 PMC123261

[32] Maffei M, Halaas J, Ravussin E, Pratley RE, Lee GH, Zhang Y, et al Leptin levels in human and rodent: measurement of plasma leptin and ob RNA in obese and weight-reduced subjects. Nat Med. 1995;1: 1155–1161. 7584987 10.1038/nm1195-1155

[33] Berglund ED, Vianna CR, Donato J Jr., Kim MH, Chuang JC, Lee CE, et al Direct leptin action on POMC neurons regulates glucose homeostasis and hepatic insulin sensitivity in mice. J Clin Invest. 2012;122: 1000–1009. 10.1172/JCI59816 22326958 PMC3287225

[34] Stanley S, Wynne K, McGowan B, Bloom S. Hormonal regulation of food intake. Physiol Rev. 2005;85: 1131–1158. 16183909 10.1152/physrev.00015.2004

[35] Tartaglia LA, Dembski M, Weng X, Deng N, Culpepper J, Devos R, et al Identification and expression cloning of a leptin receptor, OB-R. Cell. 1995;83: 1263–1271. 8548812 10.1016/0092-8674(95)90151-5

[36] Elias CF, Aschkenasi C, Lee C, Kelly J, Ahima RS, Bjorbaek C, et al Leptin differentially regulates NPY and POMC neurons projecting to the lateral hypothalamic area. Neuron. 1999;23: 775–786. 10482243 10.1016/s0896-6273(01)80035-0

[37] Hahn TM, Breininger JF, Baskin DG, Schwartz MW. Coexpression of Agrp and NPY in fasting-activated hypothalamic neurons. Nat Neurosci. 1998;1: 271–272. 10195157 10.1038/1082

[38] Schwartz MW, Baskin DG, Bukowski TR, Kuijper JL, Foster D, Lasser G, et al Specificity of leptin action on elevated blood glucose levels and hypothalamic neuropeptide Y gene expression in ob/ob mice. Diabetes. 1996;45: 531–535. 8603777 10.2337/diab.45.4.531

[39] Stephens TW, Basinski M, Bristow PK, Bue-Valleskey JM, Burgett SG, Craft L, et al The role of neuropeptide Y in the antiobesity action of the obese gene product. Nature. 1995;377: 530–532. 7566151 10.1038/377530a0

[40] Turer EE, Tavares RM, Mortier E, Hitotsumatsu O, Advincula R, Lee B, et al Homeostatic MyD88-dependent signals cause lethal inflamMation in the absence of A20. J Exp Med. 2008;205: 451–464. 10.1084/jem.20071108 18268035 PMC2271029

[41] Ginhoux F, Greter M, Leboeuf M, Nandi S, See P, Gokhan S, et al Fate mapping analysis reveals that adult microglia derive from primitive macrophages. Science. 2010;330: 841–845. 10.1126/science.1194637 20966214 PMC3719181

[42] Mori H, Hanada R, Hanada T, Aki D, Mashima R, Nishinakamura H, et al Socs3 deficiency in the brain elevates leptin sensitivity and confers resistance to diet-induced obesity. Nat Med. 2004;10: 739–743. 15208705 10.1038/nm1071

[43] Arion D, Unger T, Lewis DA, Mirnics K. Molecular markers distinguishing supragranular and infragranular layers in the human prefrontal cortex. Eur J Neurosci. 2007;25: 1843–1854. 17432970 10.1111/j.1460-9568.2007.05396.x

[44] Garbett K, Ebert PJ, Mitchell A, Lintas C, Manzi B, Mirnics K, et al Immune transcriptome alterations in the temporal cortex of subjects with autism. Neurobiol Dis. 2008;30: 303–311. 10.1016/j.nbd.2008.01.012 18378158 PMC2693090

[45] Iwamoto K, Kakiuchi C, Bundo M, Ikeda K, Kato T. Molecular characterization of bipolar disorder by comparing gene expression profiles of postmortem brains of major mental disorders. Mol Psychiatry. 2004;9: 406–416. 14743183 10.1038/sj.mp.4001437

[46] Ricciarelli R, d'Abramo C, Massone S, Marinari U, Pronzato M, Tabaton M. Microarray analysis in Alzheimer's disease and normal aging. IUBMB Life. 2004;56: 349–354. 15370883 10.1080/15216540412331286002

[47] Ibi D, Nagai T, Nakajima A, Mizoguchi H, Kawase T, Tsuboi D, et al Astroglial IFITM3 mediates neuronal impairments following neonatal immune challenge in mice. Glia. 2013;61: 679–693. 10.1002/glia.22461 23382131 PMC7165731

[48] De Souza CT, Araujo EP, Bordin S, Ashimine R, Zollner RL, Boschero AC, et al Consumption of a fat-rich diet activates a proinflammatory response and induces insulin resistance in the hypothalamus. Endocrinology. 2005;146: 4192–4199. 16002529 10.1210/en.2004-1520

[49] Holland WL, Bikman BT, Wang LP, Yuguang G, Sargent KM, Bulchand S, et al Lipid-induced insulin resistance mediated by the proinflammatory receptor TLR4 requires saturated fatty acid-induced ceramide biosynthesis in mice. J Clin Invest. 2011;121: 1858–1870. 10.1172/JCI43378 21490391 PMC3083776

[50] Posey KA, Clegg DJ, Printz RL, Byun J, Morton GJ, Vivekanandan-Giri A, et al Hypothalamic proinflammatory lipid accumulation, inflammation, and insulin resistance in rats fed a high-fat diet. Am J Physiol Endocrinol Metab. 2009;296: E1003–1012. 10.1152/ajpendo.90377.2008 19116375 PMC2681305

[51] Lai AY, Todd KG. Differential regulation of trophic and proinflammatory microglial effectors is dependent on severity of neuronal injury. Glia. 2008;56: 259–270. 18069670 10.1002/glia.20610

[52] Sofroniew MV. Molecular dissection of reactive astrogliosis and glial scar formation. Trends Neurosci. 2009;32: 638–647. 10.1016/j.tins.2009.08.002 19782411 PMC2787735

[53] Klymiuk I, Kenner L, Adler T, Busch DH, Boersma A, Irmler M, et al In vivo functional requirement of the mouse Ifitm1 gene for germ cell development, interferon mediated immune response and somitogenesis. PLoS One. 2012;7: e44609 10.1371/journal.pone.0044609 23115618 PMC3480353

[54] Brass AL, Huang IC, Benita Y, John SP, Krishnan MN, Feeley EM, et al The IFITM proteins mediate cellular resistance to influenza A H1N1 virus, West Nile virus, and dengue virus. Cell. 2009;139: 1243–1254. 10.1016/j.cell.2009.12.017 20064371 PMC2824905

[55] Alafuzoff I, Overmyer M, Helisalmi S, Soininen H. Lower Counts of Astroglia and Activated Microglia in Patients with Alzheimer's Disease with Regular Use of Non-Steroidal Anti-Inflammatory Drugs. J Alzheimers Dis. 2000;2: 37–46. 12214109 10.3233/jad-2000-2105

[56] Cameron B, Landreth GE. Inflammation, microglia, and Alzheimer's disease. Neurobiol Dis. 2010;37: 503–509. 10.1016/j.nbd.2009.10.006 19833208 PMC2823849

[57] Roundy K, Smith R, Weis JJ, Weis JH. Overexpression of RANKL implicates IFN-beta-mediated elimination of B-cell precursors in the osteopetrotic bone of microphthalmic mice. J Bone Miner Res. 2003;18: 278–288. 12568405 10.1359/jbmr.2003.18.2.278

[58] Alver A, Keha EE, Ucar F, Ovali E. The effect of carbonic anhydrase inhibition on leptin secretion by rat adipose tissue. J Enzyme Inhib Med Chem. 2004;19: 181–184. 15449734 10.1080/14756360310001650228

